# Supplementary figures and images for: Intraocular Pressure Fluctuation in Primary Open-Angle Glaucoma with Canaloplasty and Microcatheter Assisted Trabeculotomy
Source: J Clin Med. 2022 Dec 8;11(24):7279. doi: 10.3390/jcm11247279 (PMC9780827; doi:10.3390/jcm11247279)

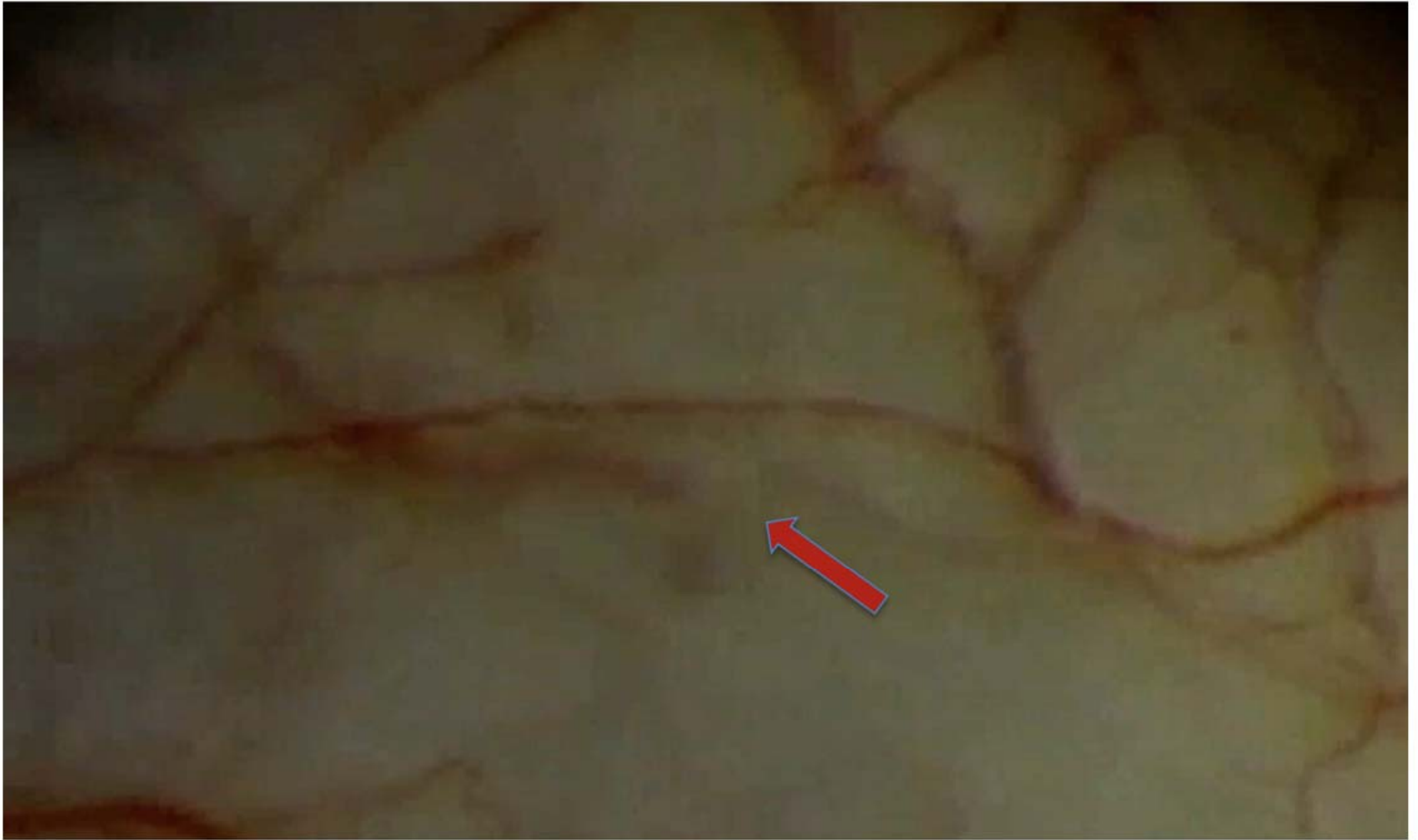

Red arrow indicates the aqueous vein

Supplement: Supplementary file 1 [file jcm-11-07279-s001.zip › Figure S1.pdf]
